# Age-related dedifferentiation of cognitive and motor slowing: insight from the comparison of Hick–Hyman and Fitts’ laws

**DOI:** 10.3389/fnagi.2013.00062

**Published:** 2013-10-10

**Authors:** Rita Sleimen-Malkoun, Jean-Jacques Temprado, Eric Berton

**Affiliations:** Institut des Sciences du Mouvement, UMR 7287, Faculté des Sciences du Sport, Centre National de la Recherche Scientifique, Aix-Marseille UniversitéMarseille, France

**Keywords:** aging, information processing, reaction time, aiming, Hick–Hyman’s law, Fitts’ law

## Abstract

The present study aimed to determine whether the general slowing hypothesis (GSH) could be extended to the motor domain by comparing cognitive and motor age-related slowing. To achieve this objective, we compared the slopes of Hick–Hyman’s law and Fitts’ law, in young and older adults. The general hypothesis was that, due to the dedifferentiation of cognitive and motor neural resources during aging, the slopes of Hick–Hyman’s law and Fitts’ law should become closer, if not similar, in older adults. Ten young adults (mean age = 26 ± 3 years) and 14 older adults (mean age = 78 ± 7 years) participated in the experiment. They had to perform a discrete rapid-aiming task and a reaction time (RT) task. In the aiming task, five index of difficulty (ID) levels were used (from three to seven bits by increments of 1.0 bit). Task difficulty was scaled via the manipulation of target distance from home position. In the RT task, five IDs were selected: 0, 1, 2, 3, and 4 bits, with incompatible S–R associations. RT and movement times were recorded. Efficiency and Brinley regression functions were calculated. Age-related slowing ratios were estimated. Response times increased in both tasks in older adults. The slopes of Hick–Hyman’s law and Fitts’ law were steeper in older adults than in young participants. In young participants, the slope of Hick–Hyman’s law was smaller than that of Fitts’ law. In older adults, no difference was found. Slowing ratios observed in both tasks were equivalent. The present results extended the GSH to the motor domain. They suggested that, due to dedifferentiation of cognitive and motor neural resources, decrease in processing speed acts as a common cause to behavioral slowing in both cognitive and motor tasks.

## INTRODUCTION

During the last decade, it has been a growing interest in aging research to explore the evolving relationship between cognitive and motor performance decline over time (Li and Lindenberger, 2002; Schäfer et al., 2006; Schaefer and Schumacher, 2010), with the underlying hypothesis that few causal mechanisms might act as pacemakers of cognitive-motor coupling (Lindenberger and Baltes, 1994; Baltes and Lindenberger, 1997). Age-related behavioral slowing, which is observed in both cognitive and motor tasks, is a good entry point in this respect since it constitutes a proxy of processing speed in the central nervous system (CNS; Salthouse, 1996, 2000; Deary et al., 2010; Eckert et al., 2010; Eckert, 2011).

In the cognitive domain, it is currently considered that behavioral slowing is mediated by a generalized deficit in processing speed of the CNS, which might be at origin of performance decline in a large variety of tasks (Birren, 1965; Birren et al., 1980; Cerella, 1985, 1991, 1994; Bashore, 1994; Salthouse, 1996; see Deary, 2000 for a discussion). In support of this hypothesis, meta-analyses using Brinley regression functions (Brinley, 1965) showed roughly constant slowing ratios between response latencies of young and older adults (i.e., 1.4–1.6), independent of the type of task (Cerella et al., 1980; Cerella, 1985). These findings supported the so-called *general slowing hypothesis* (GSH) in the cognitive domain (Cerella, 1985, 1991, 1994).

Most authors assumed that sensori-motor processing speed is irrelevant to the GSH because it is relatively spared by age-related alteration of the CNS and is included in behavioral slowing as an additive peripheral contribution (for detailed theoretical and methodological arguments, see Cerella, 1985, 1991; Bashore, 1993, 1994). This view was based on the segregation between the slowing of computational (central) and peripheral (sensori-motor) components of the cognitive tasks, which generally involve a simple motor response. However, age-related behavioral slowing is also pervasive in most motor tasks requiring complex movements (Ketcham et al., 2002; Watson et al., 2010; Mielke et al., 2012; Rey-Robert et al., 2012; Temprado et al., 2013). Since brain activity underlying programming and control of complex movements is currently considered as a computational, information-processing activity (Welford, 1977; Schmidt, 1988; Light, 1990), slowing of motor behavior presumably reflects decrease in processing speed in sensori-motor neural structures of the CNS. Thus, the question arises of whether the GSH can be extended to the motor domain. A critical question in this respect is whether behavioral slowing observed in cognitive and motor tasks dedifferentiate that is, if they become of comparable magnitude in older adults (Birren and Fisher, 1995). It might be the case because processing speed in cognitive and motor tasks becomes progressively supported by common neural resources during aging. Evidence supporting this view does exist in the literature. For instance, age-related increase in co-variation of cognitive and motor performance observed in correlation studies (Lindenberger and Baltes, 1994; Baltes and Lindenberger, 1997) suggested the existence of common neural factors to decline of both functional domains (Lindenberger and Ghisletta, 2009). This hypothesis is consistent with the pioneering observations by Birren and Botwinick (1951), who reported increased correlation (i.e., dedifferentiation) between calculation time and writing time in older adults, relative to their young participants. Recent behavioral and brain-imaging studies also showed that cognitive permeation of the motor domain becomes more accentuated during aging (Li and Lindenberger, 2002; Schäfer et al., 2006; Schaefer and Schumacher, 2010), presumably since cognitive and motor systems shared more common brain structures in older adults than in young participants (Heuninckx et al., 2005, 2008). In addition, because some of the neural underpinnings of decrease in processing speed (e.g., white matter changes) are neither specific to cognitive nor to motor areas (Penke et al., 2010; Reuter-Lorenz and Park, 2010; Eckert, 2011), one can predict to observe a generalized slowing of behavior in cognitive and motor tasks. The present study addressed this issue.

To achieve this objective, we compared response times recorded in two representative task paradigms of cognitive and motor domains that is, choice reaction time (CRT) and rapid aiming movement tasks, respectively. In this perspective, the CNS as a model human processor (MHP) composed of cognitive and motor functional sub-systems, each characterized by a specific *principle of operation* (Card et al., 1986) that is, *Hick–Hyman’s law* (Hick, 1952; Hyman, 1953) and *Fitts’ law* (Fitts, 1954; Fitts and Peterson, 1964), respectively. Hick–Hyman’s law and Fitts’ law capture the linear relationship between response time and task-related complexity variables defined in reference to quantitative theory of information processing (i.e., index of difficulty, ID in bit), in CRT and aiming movement tasks, respectively.

In reaction time (RT) tasks, the number of possible S–R associations (i.e., N alternatives) is manipulated, while the complexity of the motor response is maintained constant and minimal (Henry and Rogers, 1960; Sanders, 1990; Klapp, 1996). Thus, when low error rate is preserved in the different conditions (Pachella, 1974; Wickelgren, 1977), CRT is a reliable measure of the time needed by the CNS to reduce the uncertainty conveyed by the imperative signal. Accordingly, the index of difficulty (ID = Log_2_*Na*, ID is in bit, with Na being the number of alternatives) quantifies the amount of information (in bit) to be processed to produce a correct response. It is noticeable that RT also includes the central duration of motor response generation processes (Henry and Rogers, 1960), which are however maintained constant, minimal and independent of the duration of central processing related to response selection (Welford, 1977; Cerella, 1985; Jensen, 1987; Bashore, 1993).

Hick (1952) and Hyman (1953) showed that RT is linearly related to ID according to the following relation: RT = *a* + *b* × ID, with *a* and *b* as constants. This ID–RT linear relation – so-called Hick–Hyman’s law – reflects the *efficiency function* (EF) of information processing in the CNS. The slope of the EF is currently referred to as a measure of central processing, while the influence of peripheral factors on RT is assessed by changes in the intercept (Welford, 1984; Cerella, 1985; Bashore, 1993). Thus, the steeper the slope of Hick–Hyman’s law, the longer it takes to process a fixed amount of information by the CNS. In this respect, small slope values (30–40 ms/bit) have been currently reported in the literature, thereby suggesting that the RT task weakly loaded information-processing capacity of the CNS (Cerella, 1985; Jensen, 1987; Birren and Fisher, 1995; see below).

According to the framework of information theory, age-related slowing of RT reflects a decrease in central processing speed (e.g., Hale et al., 1987; Amrhein et al., 1991; Fozard et al., 1994; Earles and Salthouse, 1995; Salthouse, 1996; Hultsch et al., 2002). However, the effect of aging on the slope Hick–Hyman law has been scarcely described in the literature. In its extensive review, Jensen (Jensen, 1987) only referred to unpublished data (Ananda, 1985, unpublished doctoral dissertation, University of California, Berkeley) reporting a slight increase (about 10 ms/bit) in the slope of Hick–Hyman law in elderly people that is, 20–25% of the processing capacities currently observed in young adults. On the basis of a review of 11 RT studies, Welford (1984) reported estimated age-related slowing ratio to about 16% (see Cerella, 1985, for a consistent estimation). These values were smaller than those currently reported for cognitive processing speed (1.4–1.6; Cerella et al., 1980; Cerella, 1985), and close to those reported for task conditions involving weak computational requirements (1.2/1.3; Cerella et al., 1980; Cerella, 1985). A plausible explanation is that, when compatible S–R associations are used, central executive functions (EFs) associated with response selection were weakly loaded and RT tends to predominantly reflect more peripheral components (Birren and Fisher, 1995; see Yordanova et al., 2004; Falkenstein et al., 2006; Kolev et al., 2006 for supporting evidence). This raises the question of whether “pure” processing speed of the CNS (and, consequently, age-related slowing ratios) can be estimated independently of the modulating effect of EFs, which are more or less systematically involved in most cognitive tasks and are very sensitive to aging. Unfortunately, as noted by Verhaeghen and Cerella (2002), appropriate paradigms are lacking to resolve the form of the influence of executive control processes on processing speed (but see Albinet et al., 2012 for an elegant attempt in this respect). We contend that the use of incompatible S–R associations in Hick–Hyman paradigm might permit to move beyond this limitation by imposing additional load to EFs (e.g., Smulders et al., 1999; Meiran and Gotler, 2001; Eppinger et al., 2007; Vu and Proctor, 2008) and then, to get a more reliable measure of processing speed of the CNS. Indeed, it has been shown increasing S–R incompatibility – i.e., altering the natural mapping between the spatial stimulus array and the spatial response array (Fitts and Seeger, 1953; Fitts and Deininger, 1954) – significantly increased the slopes of the RT-ID EFs relative to those observed in compatible S–R associations (Welford, 1984; Sanders, 1990; see Jensen, 1987 for a review). Accordingly, Jensen (1987) recommended the use of incompatible S–R associations when assessing cognitive processing speed through Hick–Hyman’s law.

Information-processing speed can also be measured in aiming movement tasks through Fitts’ law. Fitts’ law is calculated on the basis of response times measured in rapid aiming movement tasks, consisting of moving from a home position toward a target placed at a given distance. The width (*W*) and/or distance (*D*) of the target can be varied to modulate task difficulty. In the framework of information theory, the index of difficulty (ID = Log_2_(2 × *D*/*W*), in bit) measures the amount of information to be processed to produce a fast and accurate movement in discrete and cyclic aiming tasks (Fitts, 1954; Fitts and Peterson, 1964). Accordingly, movement time (MT) was proven to be linearly related to the ID, hence to *D* and *W*, according to the following relation: MT = *a* + *B* × ID, with *a* and *b* as constants (Fitts, 1954; Fitts and Peterson, 1964). The ID–MT linear relation – so-called Fitts’ law – reflects the *EF* of information processing in the CNS to control the aiming movement. The steeper the slope, the longer it takes to process a fixed amount of information. Compared to constraints related to increasing movement accuracy (i.e., *W* manipulation), those related to movement amplitude (i.e., *D* manipulation) have been shown to result in a steeper ID–MT slope (Welford et al., 1969; Heath et al., 2011; Sleimen-Malkoun et al., 2012) that is, to globally impose greater processing demands to the information-processing system. Slopes values comprised between 60 and 100 ms/bit were currently observed in young adults, depending on whether IDs are obtained via target distance or target size manipulation (for illustrative examples, see Rey-Robert et al., 2012; Temprado et al., 2013). In addition, several studies showed longer MTs (Welford et al., 1969; York and Biederman, 1990; Haaland et al., 1993; Teeken et al., 1996; Ketcham et al., 2002) and steeper slopes of Fitts’ law (130–150 ms/bit) in older participants relative to young adults (Rey-Robert et al., 2012; Temprado et al., 2013).

In the MHP framework, Hick–Hyman and Fitts’ laws are hypothesized to quantify information-processing efficiency of cognitive and motor functional sub-domains, by expressing it under the same form (i.e., linear relations between response times and IDs) and in the same metrics (bit/s) and that, independently of the details of the mechanisms involved in RT and aiming movement tasks (Card et al., 1986). Differences between slope values currently reported in the literature on Hick–Hyman and Fitts’ laws support the hypothesis of functional separation of cognitive and motor principles of operation in young adults. Indeed, the longer time necessary to process one bit of information in aiming tasks relative to CRT tasks suggests that the sensori-motor structures are less efficient in processing information than the cognitive ones. We contend however that this conclusion could be misleading since it does not take into account the possible contamination of processing speed measured by Fitts’ law by cognitive EFs. In other words, larger slope values observed for Fitts’ law could simply reflect the fact that aiming task included a significant contribution of EFs – i.e., planning, up-dating information and inhibitory processes (see Jurado and Rosselli, 2007, for an extensive review on EFs) – which was not (or only weakly) involved in the RT task, when compatible S–R associations were used. This hypothesis is consistent with recent studies showing that EFs are currently involved in complex motor tasks (e.g., locomotion; Yogev-Seligmann et al., 2008; Verghese et al., 2010). Slowing ratios observed in aiming movement tasks (1.4/1.5; Rey-Robert et al., 2012; Temprado et al., 2013) also suggested that cognitive/executive processes are largely involved in the control of aiming movements. Indeed, these values were of comparable magnitude with those previously reported in meta-analyses of the cognitive literature, for a large variety of non-motor tasks (1.4/1.6; Cerella et al., 1981; Cerella, 1985). According to these findings, we concluded that, to test the GSH in both cognitive and motor domains, one should compare Fitts’ law with Hick–Hyman’s law calculated on the basis of response times recorded for incompatible S–R associations in CRT tasks.

The main objective of the present work was to extend the GSH to the motor domain. To achieve this objective, we compared speed of information processing measured by Hick–Hyman’s law and Fitts’ law, in young and older adults. As a prerequisite, we predicted to observe: (1) increase in the slope of the efficiency of Hick–Hyman law in young adults when using incompatible S–R associations relative to compatible ones; (2) longer response times in older adults, in both CRT and aiming movement tasks, and (3) steeper slopes for both laws in older participants. Moreover, our main hypothesis was that, due to the dedifferentiation of cognitive and motor processes during aging, aiming movement task should become more contaminated by the engagement of EF in older than in young participants. Accordingly, we predicted that the slopes of Hick–Hyman’s law and Fitts’ law should be closer (or even similar) in older adults than in young participants.

## MATERIALS AND METHODS

### PARTICIPANTS

Twenty-four right-handed subjects, separated in two age groups, participated in this experiment: 10 young adults (five men, mean age = 26 ± 3 years) and 14 older adults (seven men, mean age = 78 ± 7 years). Young participants were recruited among students of Aix-Marseille University. Older participants were recruited in a leisure and retirement club. They all lived independently and declared to be physically active. Autonomy was assessed using the six-item Katz index (Katz, 1983) and the Older American Resources and Services (OARS; Fillenbaum and Smyer, 1981) for basic (ADL) and for instrumental activities of daily living (IADL), respectively. Physical activity level was assessed using the Canadian Study of Health and Aging Risk Factor Questionnaire (RFQ; Davis et al., 2001). All participants completed a self-report to ensure that they did not suffer from cognitive or sensori-motor troubles that might bias their performance in the experimental tasks. In addition, a standardized geriatric assessment (SGA) was supervised by a medical doctor. It allowed the assessment of: (i) vision (using self-report visual functional test described by Cacciatore et al., 2004), (ii) depression (using the four-item Geriatric Depression Scale (mini GDS); Clément et al., 1997), (iii) cognition (using the clock drawing test; Shulman, 2000), (iv) pain (upper limbs or neck pain due to osteoarthritis), and (v) medication and co-morbid conditions. These assessments attested that older participants did not suffer from pathological cognitive and motor impairments. They all had their vision corrected and none of them was depressive. Twelve participants of the elderly group were practicing a regular physical activity, consisting in walking for at least 30 min three times per week; the remaining two participants walked one time per week for at least 30 min. For ADL, 13 participants had a maximum score (6/6) and one had a score of 5/6. For IADL, 12 participants had a score of 14/14, one had a score of 13/14 and one a score of 12/14. None of the older participants presented any deficits leading to their exclusion from the study. Informed consents to participate in the study were obtained from all young and older participants. None of them had a prior experience with the experimental tasks. The protocol was approved by the local ethic committee of Aix-Marseille University, and has therefore been in accordance with the ethical standards laid down in the Declaration of Helsinki.

### APPARATUS AND TASK

Participants were seated in an adjustable height chair at a table in a bright room and no noise disturbance. They had to perform a discrete rapid-aiming task (Fitts’ task) and a RT task (Hick–Hyman’s task). The order of presentation of these two tasks was counterbalanced.

#### Aiming task

The task consisted in making home-to-target aiming movements with the right arm by sliding a hand-held non-marking stylus (Wacom, Generation 2 tip sensor) over the surface of a Wacom graphic tablet (Intuos4 XL) placed on the tabletop directly in front of the participants (portrait orientation). The home position was marked by a black square (5 mm × 5 mm) and the target was symbolized by a black horizontal rectangle (40 mm × 7 mm). They were both printed on a white paper sheet and inserted under the tablet’s transparent plastic film cover. Home position and target center were aligned and located at 14.5 cm from the left side of the tablet’s sensitive area. Sliding movements were performed in the anterior–posterior direction and were executed with a combination of shoulder flexion and elbow extension. Participants were instructed to constantly keep their back against the chair support to prevent trunk compensations. The graphic tablet was connected (via a USB port) to a portable PC (Dell, Latitude D420). A customized software was used to acquire and save the kinematic data generated by the displacements of the stylus on the tablet with a sampling frequency of 250 Hz.

#### Reaction time task

The RT task was performed on a response console similar to “Jensen’s box” (1987). The console consisted of a metal panel (360 mm × 400 mm × 4 mm) tilted at a 30° angle. A home capacitive touch sensor switch (CSE 16, Schurter, diameter: 20 mm) was located at the lower center of the panel. It was surrounded by 16 equidistant (17°) similar buttons arranged in a semi-circle, on an arc of 255°, with a radius of 8 cm. All buttons were screwed on the metal plate. Each response button on the panel was associated with a red and a green flat LED (Agilent Technologies, diameter: 5 mm). The red LED was used for pre-cuing the potential responses; the green LED was used to indicate the effective response that is, the button to reach. Three centimeters above the central home button were placed three yellow flat LEDs (Agilent Technologies), which were used as warning signals for the preparation period. The ignition sequence of the LEDs was the following: (1) pre-cuing of response alternatives (red LED, duration: 2 s), (2) preparatory period of 1000, 1250, 1500, or 1750 ms presented randomly (yellow LEDs blanking three times); and (3) response signal (green LED staying on until reaching of the response button). Correspondingly, participants held their right index finger on the home button, then, at the onset of the imperative stimulus (IS), they were instructed to move as fast as possible and press the corresponding response button (see below for description of compatible and incompatible S–R association conditions).

An acquisition card (NI USB 6608) was used to record the data and another one (NI USB 6501) to control the LEDs by managing digital inputs and outputs. Both cards were connected to a laptop (Dell, Latitude D420) via USB ports. The whole display was controlled by an interface developed under LabVIEW (version 10.0, National Instruments), which allowed the experimenter to start the ignition sequence of the LEDs.

### PROCEDURE

#### Aiming task

Experimental conditions consisted of five ID levels, ranging from three to seven bits by increments of 1.0 bit. Task difficulty was scaled via the manipulation of target distance from home position. Five distances were used: 28 mm (ID3), 56 mm (ID4), 112 mm (ID5), 224 mm (ID6), and 448 mm (ID7). At the start of each trial, the stylus was placed on the home position. Participants were instructed to preserve optimal speed–accuracy trade-off that is “to move as fast as possible from the starting position toward the target and to stop on it without making any (overshoot or undershoot) errors.” For each of the five ID conditions, participants were allowed three unrecorded practice trials then requested to complete a block of 16 trials. The order of presentation of the conditions was randomized in-between participants. In order to help participants to adjust the adopted speed–accuracy trade-off, the experimenter provided verbal feedbacks after each condition. In each ID condition, the allowed error rate was 12.5% (maximum 2 trials out of 16). If more than two trials were missed, the missed trials were repeated at the end of the condition. In this respect, one young participant had to repeat three trials at ID4, and another four trials at ID6. Only one older participant repeated three trials at ID7.

#### Reaction time task

A pilot experiment was carried out with a group of six young participants (25–30 years) with three IDs (0, 1, and 3 bits) to verify the conformity of RT data to Hick–Hyman’s law and to determine the most appropriate experimental conditions of S–R compatibility. These participants were not included in the subsequent experiment.

In the pilot experiment, we conformed to the procedure recommended by Jensen (1987) and the results were similar to those reported in his review. Specifically, they confirmed that: (1) both individual and collective (group mean) RT data followed Hick–Hyman’s law, (2) RT did not depend on the response button position on the panel, and (3) learning effect on RT did not occur for a small number of trials (<30). In addition, we tested the effect of S–R compatibility on RT (Fitts and Seeger, 1953). In incompatible conditions, participants were requested to reach the response button opposite to the button cued by the green LED, with respect to the central symmetry axis of the button arrangement. Results showed that the slope value of the EF (31 ms/bit) was the same as that calculated by Jensen (1987) for a sample of nine studies in young participants (27 ms/bit). Similar values were also observed for the intercepts (300 ± 15 and 270 ms, respectively). In incompatible conditions, RTs also followed Hick–Hyman law but the slope of EF (RT–ID relationship) increased (68 ms/bit) and approached those observed for Fitts’ law in previous studies (Rey-Robert et al., 2012; Temprado et al., 2013). Thus, as predicted, incompatible S–R associations increased the slope of Hick–Hyman law, presumably by loading central EF associated with response selection.

In the subsequent experiment, five levels of difficulty of incompatible S–R associations were selected: ID = 0, 1, 2, 3, and 4 bits, which corresponded to 1, 2, 4, 8, and 16 possible responses. It is noticeable that, due to task specificities, difficulty levels in both tasks were different. Our objective was not to compare similar IDs in the two tasks. It did not preclude however the comparisons between the slopes of Hick–Hyman and Fitts’ laws.

Participants had to react as quickly as possible and move their finger toward the button that was opposite to the one corresponding to the lightened green LED, with respect to the central symmetry axis of the panel. Sixteen trials were carried out in each ID condition. Following the recommendations provided by Jensen (1987), the ID conditions were always presented in the same order to compare the differences between groups. Accordingly, the order of presentation was: 3/0/1/4/2 bits. The locations of effective responses on the button panel were also balanced across trials so that all the different locations were used in each ID condition. To encourage participants to perform the task as quickly as possible, participants were informed of their total response time (TR + TM) after each trial. Trials in which participants anticipated the response signal (<100 s), moved in the wrong direction, or missed the target button, were considered as response errors. In each ID condition, the allowed error rate was 12.5% (maximum 2 trials out of 16). If in any ID condition more than two trials were missed, these trials were repeated. This was the case for only one young participant who had to repeat three trials at ID4.

### VARIABLES AND DATA PROCESSING

#### Aiming task

The pen-tip raw data were filtered using a second-order dual pass (no phase-lag) Butterworth filter with a cut-off frequency of 10 Hz. Time series of position and velocity profiles were then computed. Movement onset and offset were determined on the basis of velocity profiles using the *optimal algorithm* of Teasdale et al. (1993). The critical velocity threshold was obtained by multiplying peak velocity by 0.04. MT, defined as the elapsed time between movement onset and offset, was then computed.

#### Reaction time task

Reaction times and MT were recorded. RT was defined as the time elapsing between the lighting of the green LED and the release of the home button. RT values above three times the standard deviation (SD) were discarded from the analysis. MT was defined as the time elapsing between the release of the home button and the touch of the response button. Participants were instructed to respond as fast as possible but they were not informed that RT and MT were recorded and analyzed separately.

### STATISTICAL ANALYSES

Two-way ANOVA (group × ID) with repeated measures on ID has been carried out on all variables. The sphericity of the data was verified for each analysis with the test of Mauchley and, in case of violation, the Greenhouse–Geisser correction was applied to the degrees of freedom (df). Accordingly, the reported df correspond to the nearest whole number. The effect size was calculated as: η^2^=SS explained/SS total. *Post hoc* analyses were carried out using Newman–Keuls test.

To compare response times observed in CRT and aiming movement tasks, EFs and Brinley functions (BF) were calculated. EFs quantified the relation between the *ID* and the temporal variables recorded in each task (RT and MT). They were determined by using linear regressions carried out on mean group values in each task. EFs representing Fitts’ law were conducted on *MT* data and those representing Hick–Hyman’s law on *RT* data. Linear regressions of BF (Brinley, 1965) were calculated after plotting mean values of *MT* and *RT* observed in young participants (abscissa) against those observed in elderly (ordinate). Thus, it resulted in two BF: one for Fitts’ task and one for Hick–Hyman task.

Efficiency and BF differed in their purpose and, hence, were complementary. By comparing between the slopes of the different EFs, we assessed information-processing capacities in each age group (in bit/s). BFs, on the other hand, allowed the estimation of age-related slowing ratios, which were measured by the slope values of the regression functions. The comparison between the calculated slopes in each task allowed determining if sensori-motor and cognitive processes presented the same slowing ratios with aging. In all regression analyses Student’s *t*-statistic was used to compare between slopes. For all statistic tests the used threshold of significance was 0.05.

## RESULTS

Mean and SDs values of response times observed in each ID level along with the statistics of ANOVA are summarized in **Table 1**.

**Table 1:** Mean and standard deviation values of response times and ANOVA results.

ID	Young *M* (SD)	Elderly *M* (SD)	ANOVA results			
			Effect	DF	*F*	reta^2^
**MT Fitts’s task**
3	228 (48)	387 (122)				
4	296 (64)	460 (103)	Group***	1.22	23.13	0.20
5	401 (55)	604 (138)	ID***	2.47	324.9	0.55
6	500 (34)	746 (172)	Group × ID***	2.47	9.56	0.02
37	652 (47)	980 (201)				
**MT Hick–Hyman’s task**
0	201 (54)	296 (62)				
1	195 (55)	289 (61)	Group***	1.22	20.11	0.31
2	218 (54)	331 (91)	ID	1.32	3.07	0.04
3	239 (60)	370 (89)	Group × ID	1.32	0.52	0.007
4	237 (56)	379 (106)				
**RT Hick–Hyman’s task**
0	308 (37)	413 (58)		**		
1	384 (62)	526 (84)	Group***	1.22	32.62	0.21
2	493 (90)	638 (75)	ID***	2.43	122.8	0.28
3	569 (85)	808 (144)	Group × ID***	2.43	10.39	0.04
4	600 (98)	946 (191)		**		

*M*, mean; SD, standard deviation; *p < 0.05; **p < 0.01; ***p < 0.001.

### AIMING MOVEMENT TASK

#### Analysis of variance on movement time

The ANOVA carried out on MTs revealed a main effect of group [*F*_(1,22)_ = 23.13, *p* < 0.001, η^2^ = 0.2], with older participants being slower, and of ID [*F*_(2,47)_ = 324.93, *p* < 0.001, η^2^ = 0.55]. It also showed a significant group × ID interaction [*F*_(2,47)_ = 9.56, *p* < 0.001, η^2^ = 0.02]. *Post hoc* decomposition of the interaction showed that MT increased with ID for both groups (*p* < 0.001). In addition, MTs were longer in older adults and the difference between the two groups was larger for the higher difficulty level (ID7, *p* < 0.05).

#### Efficiency function

**FIGURE 1:**
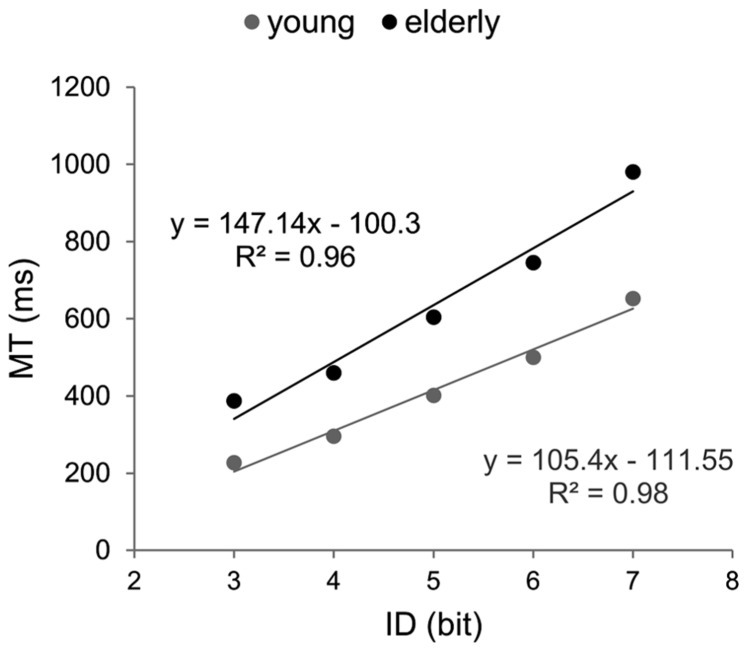
**Efficiency functions for movement time in Fitts’ task.** Young participants’ data and linear regression estimates are presented in gray and those of older participants in black.

Linear fittings of ID–MT relation in each age group, along with the corresponding equations, can be found in **Figure 1**. Fitts’ law fitted well MT data in both groups (R_young_^2^ = 0.98; R_elderly_^2^ = 0.96). However, EF of the group of older participants presented a significantly steeper slope (147 vs. 105, *p* < 0.05), with no significant difference between the intercepts (-100.3 vs. -111.5, *p* > 0.05).

### CHOICE REACTION TIME TASK

#### Analysis of variance

The ANOVA carried out on RTs showed main effects of group [*F*_(1,22)_ = 32.62, *p* < 0.001, η^2^ = 0.21], and ID [*F*_(2,43)_ = 122.83, *p* < 0.001, η^2^ = 0.28], with a group × ID interaction [*F*_(2,43)_ = 10.39, *p* < 0.001, η^2^ = 0.004). Older participants were slower than young participants with a significant inter-group effect for ID3 and ID4 (*post hoc* decomposition, *p* < 0.001). The ID significantly increased RTs with significant inter-conditions differences for both groups (*post hoc* decomposition, *p* < 0.01) except for ID3–ID4 in the young group (*p* > 0.05).

We also analyzed MTs associated with RTs. Results showed a main effect of age [*F*_(1,22)_ = 20.11, *p* < 0.001, η^2^ = 0.31], but no significant effect of ID [*F*_(1,32)_ = 3.07, *p* > 0.05, η^2^ = 0.04], nor a significant group × ID interaction [*F*_(1,32)_ = 0.52, *p* > 0.05, η^2^ = 0.007]. Hence, older participants were slower than young participants. In addition, for both young and older participants, MT was not significantly changed across the ID levels.

#### Efficiency functions

**FIGURE 2:**
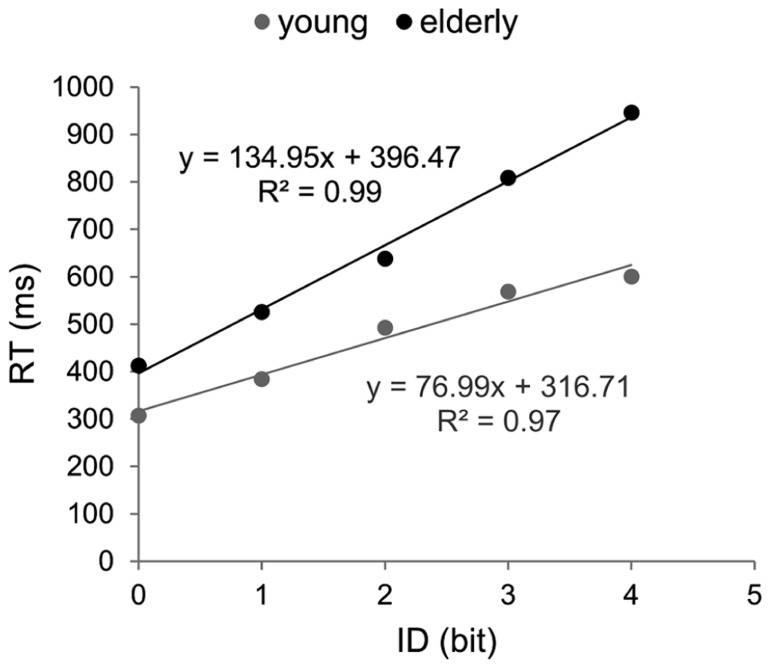
**Efficiency functions for reaction time in Hick–Hyman’s task.** Young participants’ data and linear regression estimates are presented in gray and those of older participants in black.

Efficiency functions were estimated for mean RTs in each group (**Figure 2**). Coefficients of determination were higher than 95% (R_young_^2^ = 0.97; R_elderly_^2^ = 0.99). The EF of older participants showed a significantly steeper slope (134.9 vs. 76.9, *p* < 0.001) and a greater intercept (396.5 vs. 316.7, *p* < 0.01) compared to those observed for young participants

Since the results of the ANOVA showed that RT did not significantly increase after ID4 in the young group, ID–RT relation was re-evaluated for the difficulty range between zero and four bits (R_young_^2^ = 0.99; R_elderly_^2^ = 0.99). The exclusion of ID4 increased the slope of the young’s EF but it remained significantly inferior to the older one (89 and 134.9, respectively, *p* < 0.001).

### COMPARISON BETWEEN FITTS’ LAW AND HICK–HYMAN LAW

#### Efficiency functions

We compared the slopes of EFs calculated for Hick–Hyman and Fitts’ laws in each group of participants. Results showed that the slope of Fitts’ law was significantly larger than that of Hick–Hyman’s law in young participants, independent of whether three or four IDs were considered in the CRT task (105.4 vs. 77 and 89, respectively, *p* < 0.05). Conversely, the slopes of Fitts and Hick–Hyman’s laws observed in older adults did not differ from each other (147.1 and 134.9, respectively, *p* > 0.05). As for the intercepts, in both groups, Hick–Hyman’s law presented the largest values (*p* < 0.001).

#### Brinley functions

**FIGURE 3:**
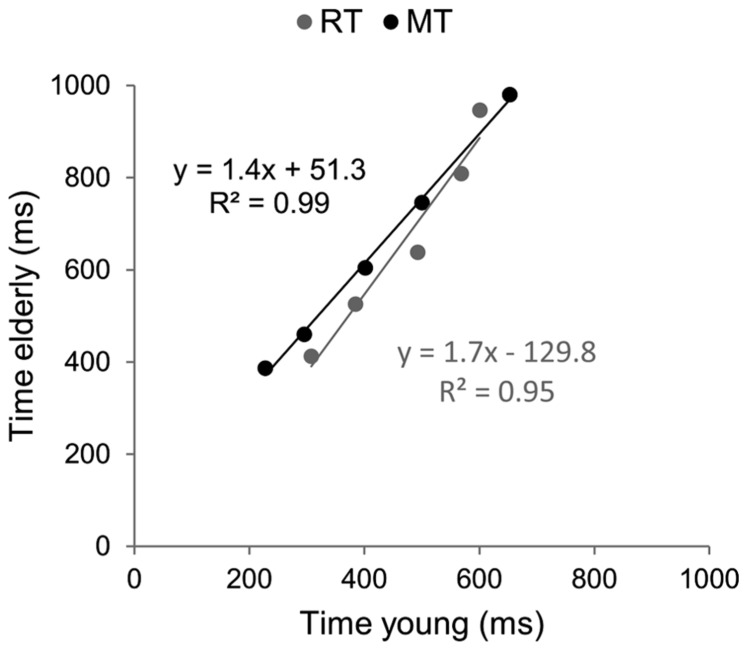
**Brinley plots. Data and linear regression estimates of reaction time (observed in Hick–Hyman’s task) are presented in gray and those of movement time (observed in Fitts’ task) are presented in black.** Each data point corresponds to an ID condition (from the easiest to the hardest).

**FIGURE 4:**
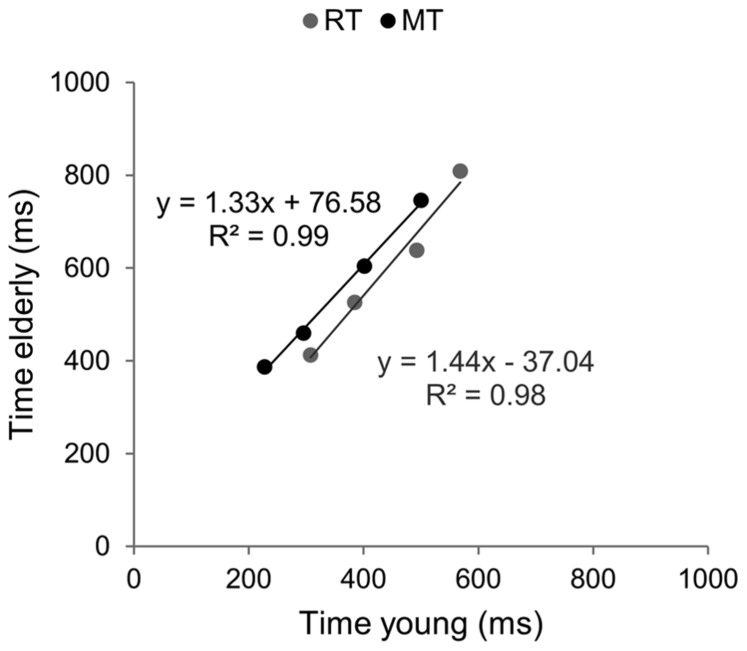
**Brinley plots without the hardest ID condition.** Data and linear regression estimates of reaction time (observed in Hick–Hyman’s task) are presented in gray and those of movement time (observed in Fitts’ task) are presented in black. Data points correspond to the first four ID conditions of each task, respectively.

Brinley functions were calculated for MT and RT, allowing a quantification of age-related changes in performance in each task, which was then compared. The estimates of BFs are reported in **Figure 3**. For both tasks the slopes of BFs were significantly different from 1 (1.4 for the aiming task and 1.7 for the CRT task, *p* < 0.001). However, although the slope of BF calculated in the cognitive task was 20% larger than that observed in the motor task, they were not significantly different from each other (*p* > 0.05). When the analysis was conducted between ID0 and ID4 the inter-group difference in slope was reduced (1.33 for MT with *R*^2^ = 0.99, and 1.44 for RT with *R*^2^ = 0.98; see **Figure 4**).

## DISCUSSION

One of the most reliable findings in aging literature is that older adults respond more slowly than younger adults in both cognitive and motor tasks. Hence, several studies attempted to quantify slowing ratios between response latencies of older and younger adults in cognitive tasks (e.g., Cerella et al., 1980; Cerella, 1985) and, more recently, in rapid aiming movement tasks (Rey-Robert et al., 2012; Temprado et al., 2013). However, until now, it had never been explored whether similar slowing ratios could be observed in cognitive and motor domains, in the same group of participants. It could be the case because, due to the dedifferentiation of neural information-processing resources during aging, the two operation principles presumably related to separate cognitive and motor sides of the MHP in young adults become related to one another in later life (Lindenberger and Baltes, 1994; Birren and Fisher, 1995; Lindenberger and Ghisletta, 2009). The present experiment addressed this issue in young and older adults by comparing Hick–Hyman and Fitts’ laws.

Results observed in the pilot experiment of the present study confirmed that, in the CRT task, the use of incompatible S–R associations led to steeper slope of Hick–Hyman’s law. This result was consistent with those reported by Jensen (1987) and confirmed our prediction with respect to the effect of load imposed to EFs on cognitive processing speed. This result did lend credence to the strategy used in the present study to investigate the GSH. Specifically, it consisted of assessing processing speed in a cognitive task where EFs were strongly involved to further compare it with that observed in a motor task, also involving EFs. This strategy is different from those consisting of isolating the contribution of EF and processing speed since they are hypothesized to be the two critical and separate mediators of age-related decline of performance in a wide range of tasks (Verhaeghen and Cerella, 2002; Lindenberger and Ghisletta, 2009; see Albinet et al., 2012) for an elegant contribution in this respect). Our results are however consistent with the hypothesis that “pure” cognitive processing speed is rather difficult to quantify in isolation since it is often more or less contaminated by the involvement of EF, even in most cognitive tasks (see Cerella, 1985; Salthouse, 1996 for a converging point of view). Thus, using incompatible S–R associations in the choice reaction task allowed estimating a more specific measure of cognitive processing speed than the use of compatible S–R conditions (see Jensen, 1987 for a converging point of view).

### EFFECTS OF AGING ON HICK–HYMAN’S LAW AND FITTS’ LAW

As a prerequisite, we explored the effects of aging on Hick–Hyman’s law and Fitts’ law, thanks to the use of a large range of ID values in both RT and aiming movement tasks.

Results showed that RTs perfectly followed Hick–Hyman law in both young and older adults. An exception to the linear increase in response time with ID was noticed in the RT task, for young participants, between ID3 (eight S–R pairs) and ID4 (16 S–R pairs). Such discontinuity was not observed in older adults. In addition, aging resulted in steeper slope and slightly greater intercept of Hick–Hyman law, thereby suggesting that central components were more loaded by incompatible S–R associations than peripheral ones (Welford, 1984). The analysis carried out on MTs showed that older adults were significantly slower than young participants. On the other hand, MTs were not affected by ID increase. This result showed that participants did not strategically trade RT and MT to make a part of the decision while moving toward the endpoint target, when ID increased (Jensen, 1987).

In Fitts’ task, results observed for MTs were consistent with those observed in our previous studies (Rey-Robert et al., 2012; Temprado et al., 2013). Indeed, regression functions showed that aging resulted in steeper slopes of Fitts’ law in older adults. It is noticeable that intercepts were larger in older adults than in young participants. This result is not surprising however; it suggests that musculo-skeletal peripheral factors were also affected by aging, thereby lengthening additively MTs (Allen et al., 2004).

Overall, as predicted: (1) response times were significantly lengthened in older adults in both the CRT and aiming movement tasks, and (2) difference of response times observed between young and older adults increased with ID in both the cognitive and motor tasks, thereby revealing a previously described *age-complexity effect* (Birren, 1965; Cerella et al., 1980; Cerella, 1985). These results suggest that processing speed decreased in a quantifiable amount (in bit/s) in older adults, in both the RT and aiming movement tasks. This hypothesis was confirmed by the analysis of slowing ratios.

### AGE-RELATED SLOWING RATIOS IN REACTION TIME AND AIMING MOVEMENT TASKS

Brinley functions allowed determining whether behavioral slowing observed in CRT and aiming movement tasks were of comparable magnitude, independently of whether the slope of Hick–Hyman’s law and Fitts’ law were different or not. Indeed, it could be that the effects of aging would be of comparable magnitude, even if the underlying neural resources involved in the two tasks were different. Brinley regression functions indicated equivalent slowing ratios for MT and RT (1.3–1.4). These results confirmed that simple mathematical (linear) functions can predict the latencies of older adults from the latencies of younger participants, independent of the details of information-processing mechanisms involved in CRT and aiming movement tasks. The comparison of magnitude of the slowing ratios observed for RT and MT and those reported by Cerella et al. (1980) suggest that mental information-processing resources were significantly involved in both tasks. Indeed, in their review, Cerella et al. (1980) analyzed 18 studies that included a wide range of information-processing tasks and reported a mean slowing ratio of 1.36. However, they also noticed a smaller ratio (1.15) when only sensori-motor tasks were considered, thereby suggesting that these tasks were relatively unaffected by aging because they only weakly loaded the information-processing resources that are altered in the aging brain (Kail, 1986, 1988). Accordingly, slowing ratios observed in the present study suggested that: (i) computational components were strongly involved in both RT and aiming movement tasks, and (ii) aging similarly affected information-processing speed in both tasks. If one accepts the classic hypothesis of functional separation of cognitive and motor domains, this result was rather unexpected. The comparison between Hick–Hyman law and Fitts’ law allowed testing the dedifferentiation hypothesis, which might explain the equivalent slowing ratios observed in both tasks.

### COMPARISON OF HICK–HYMAN LAW AND FITTS’ LAW IN YOUNG AND OLDER ADULTS

Results observed in young adults showed that the slopes of Hick–Hyman’s law and Fitts’ law were significantly different. Specifically, the slope of Fitts’ law was larger than those of Hick–Hyman law. One can conclude from this result that neural information-processing resources were more loaded in aiming movement task than in CRT task. In addition, the difference between the slopes of the two laws strongly suggested that, in the aiming movement task, the lower processing speed reflected the conjunction of constraints imposed to cognitive EFs and sensori-motor mechanisms while, in the CRT task, processing speed prominently reflected the efficiency of EFs with minimal influence of sensori-motor mechanisms (Cerella, 1985; Jensen, 1987). Thus, the results observed in young adults are consistent with the MHP, namely that cognitive and motor sides are governed by similar but functionally separated operation principles (Hick–Hyman’s law and Fitts’ law), which rely on different information-processing resources in the CNS.

As predicted, results observed in older adults were different. Indeed, contrary to young adults, in the elderly, no significant difference was observed between the slopes of Hick–Hyman’s law and Fitts’ law. This result supported the GSH that is, the existence of a general limitation of processing speed in the aging brain, which acts as a common cause to behavioral slowing in RT and aiming movement tasks. According to the dedifferentiation hypothesis, a plausible explanation is that, with age, neural resources involved in CRT and aiming movement tasks become less specific and aiming movement task engage a compounded system in which cognitive and motor resources are closely intertwined. Possible candidates in this respect are frontal structures, which are known to be involved in numerous functions, including response selection and movement control (Stuss and Benson, 1983, 1984; Bashore, 1993; Schretlen et al., 2000). Accordingly, because frontal structures might be more and more involved in the control complex movement tasks during aging (Heuninckx et al., 2005, 2008; Yogev-Seligmann et al., 2008), age-related structural and functional alterations of frontal lobes might mediate changes of comparable magnitude in processing speed in both cognitive and motor tasks (Bucur et al., 2008; Eckert et al., 2010; Eckert, 2011). Of course, in the lack of detailed exploration of brain activity, evidence of neural dedifferentiation was indirect and only supported by the comparison of slopes of Hick–Hyman’s law and Fitts’ law that is, by the equivalent slowing ratios observed in CRT and aiming movement tasks.

## CONCLUSION AND PERSPECTIVES

The present study investigated, for the first time to our knowledge, the relationship between cognitive and motor aging in the framework of MHP (Card et al., 1986). To achieve this objective, two principles of operation were compared (i.e., Hick–Hyman law and Fitts’ law) in the same groups of participants, each presumably indexing information-processing speed in cognitive and motor tasks, respectively. Results confirmed that, under the reserve that incompatible S–R associations were used in the CRT task, the slopes of Hick–Hyman law and Fitts’ law became closer in older adults than in young adults. This result provided a direct evidence of age-related co-variation of behavioral slowing in the cognitive and motor domains, as a result of unspecific limitation of processing speed in the CNS. It also extends to the motor domain previous theoretical positions that assumed that behavioral senescence is a relatively low dimensional process, in which a small number of causal factors determine performance decline in a wide variety of tasks (e.g., Lindenberger and Baltes, 1994; Baltes and Lindenberger, 1997; Lindenberger and Ghisletta, 2009). However, in the present study, we used the same behavioral marker to assess dedifferentiation of cognitive and motor domains (i.e., processing speed), instead of comparing different performance variables (e.g., grip strength and cognitive speed), as in classic correlation studies (e.g., Anstey and Smith, 1999). Thus, from a methodological perspective, the present study introduced a theoretically grounded approach to investigate processing speed as a common cause to cognitive and motor slowing. The proposed strategy was also different from those currently used in the studies investigating cognitive–motor coupling in motor tasks, which generally consisted of correlating performance in assessment tests of EFs (e.g., Trail Making Test) with motor performance (see Yogev-Seligmann et al., 2008 for an illustrative example in locomotion). Here, inspired from Salthouse (Salthouse, 1994, 1996) and Cerella’s (Cerella, 1985, 1991; Cerella and Hale, 1994) theoretical and empirical work, we assessed processing speed in tasks involving a strong engagement of EFs while differing in their fundamental nature (cognitive and motor). By doing that, the objective was not to study processing speed and EFs in isolation but rather, to assess the relative contribution of EFs and motor neural resources to processing speed, in the context of the their interaction.

The findings of the present study might have potential clinical applications. Indeed, they suggest that Hick–Hyman’s law and Fitts’ law, which appear to be critical markers of age-related decrease in processing speed, could be used as simple tests of the status of the CNS with respect to processing capacities. It remains however to determine whether slowing ratios observed in the present study are similar in a wide range of motor tasks (e.g., locomotion, postural oscillations⋯). In addition, the present approach might open new perspectives to investigate the effects of training on processing speed. Specifically, the question arises of whether extensive practice in one task could induce a decrease in processing speed in the other task (aiming to RT task and vice versa). Studies recently done in our group to address this issue showed encouraging results in this respect (Temprado, 2012; Decker and Temprado, in preparation).

## Conflict of Interest Statement

The authors declare that the research was conducted in the absence of any commercial or financial relationships that could be construed as a potential conflict of interest.
